# Origami-inspired systems that improve adult diaper performance to enhance user dignity

**DOI:** 10.1017/wtc.2021.17

**Published:** 2022-05-11

**Authors:** Nathan C. Brown, Hunter T. Pruett, Diana S. Bolanos, Corinne Jackson, Bridget Beatson, Spencer P. Magleby, Larry L. Howell

**Affiliations:** Department of Mechanical Engineering, Brigham Young University, Provo, Utah, USA

**Keywords:** Absorbent clothing, adult diaper, incontinence, liquid activated shaping, self-stiffening, compliant mechanism, deployable mechanism

## Abstract

This paper proposes a novel origami-inspired adult diaper design that improves discretion by reducing sag and increasing wicking across the entire diaper pad. While other diapers rely on supporting elastics to reduce the sag of the diaper as a whole, this paper proposes an absorbent core that uses liquid activated shaping to take a specified shape. Origami-based folds are also incorporated into the diaper design to increase wicking performance. The paper introduces a disposable compliant mechanism waistband used to deploy the diaper, making it easier to put onto one’s body.

## 1. Introduction

Diapers have been traditionally used to alleviate the effects of incontinence in users from all demographics. Many companies and medical researchers have worked to create new technologies to improve adult diapers to better suit the needs of their users. Current diaper solutions include absorbent liners, perineal pads, reusable absorbent underwear, and disposable adult diapers (Abrams et al., 2013; DeMarinis et al., 2018; Davis and Wyman, 2020). Disposable adult diapers include both traditional tab and pull-up styles. Disposable diapers have been enabled through cost-effective materials, efficient manufacturing technologies, and the implementation of superabsorbent polymers (SAP). SAP is used to absorb and retain moisture, increasing diaper absorbency, and reducing leaking. In a dry diaper, SAP is a coarse powder with grains similar in size and texture to salt. When added to water, the grains swell in size as the liquid is absorbed into the SAP. These advances in diaper technology work to improve the specific issues of discretion, wicking, sag, and usability of absorbent diaper designs.

Incontinence is the loss of either urinary or bowel control, varying from slight loss to complete loss of control (Haylen et al., 2010). Urinary incontinence can affect all ages, but is most common in older aged adults (Johannesson et al., 1997), women after childbirth (Gyhagen et al., 2019), and men after prostate surgery (Shamliyan et al., 2009; Tienza et al., 2018).

While urinary incontinence is common, it is surrounded by a negative social stigma (Siddiqui et al., 2014; Southall et al., 2017; Devendorf et al., 2020). Individuals with incontinence are affected in many ways including loss of dignity, decreased socialization, increased depression and stress, and self-consciousness that has adverse effects on quality of life and productivity (Milsom et al., 2013). The use of adult diapers can be a source of embarrassment and perceived loss of control or independence. Ad campaigns specifically target this consumer challenge by focusing on product secrecy and discretion. To a user, a discreet diaper means a less stressful overall experience, and less impact on their lifestyle.

Not only can diapers have an emotional effect on individuals, they can also provide a physical burden to less mobile users. Diapers are typically compressed in packaging to reduce shipping and packaging costs. The absorbent core in many diapers consists of a fluffy wood pulp/SAP matrix, which stiffens when compressed. Once stiffened into the compressed state, the diaper is more difficult to open and put on. For individuals with limited mobility, it can be very difficult to maneuver feet into and through a compressed diaper, even while sitting. In addition, it can even be difficult for these individuals to use both hands to stretch the diaper open while simultaneously maneuvering their feet through the compressed diaper. These difficulties highlight the need for functions that assist the user in opening or unfolding the compacted diaper.

Discretion in a diaper is a function of its shape and thickness when dry and the amount of sag when wet. Sag is often accentuated when liquid pools into the bottom of the diaper resulting in localized swelling. It is proposed that by distributing the fluid more evenly throughout the entire diaper, diaper sag can be reduced.

Innovative technologies introduced in this paper could lead to improved diaper discretion while maintaining high-absorbance performance. This paper outlines origami-inspired concepts for a sag-reducing absorbent core, a liquid distribution layer that improves wicking, and a self-deploying diaper waistband that makes it easier to put on. These concepts are then implemented into a fully integrated diaper design which will herein be referred to as the “CMR Diaper,” named after the Compliant Mechanisms Research group. This designs improves diaper performance and discretion, which could lead to improved diaper usability.

This paper is organized as follows. Section 2 defines and discusses the challenges associated with designing a disposable diaper and the current technologies to address those challenges. Section 3 introduces three subsystem designs that advance the technological solutions. This includes designs for an absorbent core, wicking layer, and deployable waistband. Testing methods and results for each subsystem are presented. Section 4 presents the integration of the same subsystems into a fully integrated diaper. Testing methods and results for the integrated diaper are presented. Lastly, Section 5 contains conclusions and discussion.

## 2. Background

To better understand the technologies that will be discussed, we review the major challenges of current diaper designs including diaper discretion, sag, wicking performance, and ease of putting diaper on. In addition, a brief review of origami and origami-inspired design are discussed.

### 2.1. Discretion

The crease lines and folds of many diaper can be seen through a user’s clothing. This happens when the diaper is dry, however, the effect is accentuated when the diaper is wet. Wetting causes sag, which can also be seen through a user’s clothing. A discreet diaper should be not be noticeable when either dry or wet.

Diaper manufacturers typically achieve discretion by using light or conforming fabrics, using materials that are not noisy, and choosing color schemes and textures that mimic nonabsorbent undergarments. Additional technology is needed to improve the indiscretion caused by creasing and sag. Implementing a thinner absorbent core would improve discretion when dry, and working to reduce sag would improve discretion when wet.

### 2.2. Sag

Current diaper designs use elastic bands called gathers around the waist and leg to conform to the body. Gathers effectively form the edges of the diaper to the body, but do little to form the bulk of the diaper to the body when the diaper is wet. Once wet, there is little in the diaper to support the newly soaked pad. As a result, the entire diaper structure sags downward, pulling away from the body. The swelling and sagging of the diaper may create a source of discomfort for the user and can be noticeable to others through the user’s clothing and gait.

Sag is caused by the downward weight of the unstructured, saturated absorbent core. Some products may address sag through the use of fabrics with anisotropic stiffness. Such anisotropy allows the fabric to stretch around the user’s waist but not in the direction of sag (in the sagittal plane). While this begins to give support to a saturated absorbent core, the shape of the sagging pad is still present. Additional structuring of the absorbent core in conjunction with existing anisotropic material would significantly decrease product sag, enhancing the user’s experience.

### 2.3. Wicking

Current incontinence products use SAP to rapidly absorb fluids. SAP can absorb between 30 and 60 times its original mass when in a 0.9% saline solution (Dey et al., 2016; Sultana et al., 2018; Kakonke et al., 2019). This also means that as the SAP absorbs the liquid, it grows in size. In a diaper, localized swelling can occur when urine is not evenly distributed through the absorbent core. Such localized swelling causes the diaper to sag. Distributing the fluid evenly throughout the entirety of the pad decreases the localized expansion of the pad, thereby reducing sag.

Wicking can be defined as the spontaneous absorption of a liquid into a porous medium by the action of a capillary flow (Sampath and Senthilkumar, 2009; Masoodi and Pillai, 2010), and has been shown to be an essential part of maintaining user comfort in clothing (Sampath and Senthilkumar, 2009), including incontinence products (Abrams et al., 2013).

Currently, most incontinence products utilize a single layer of nonwoven synthetic material for wicking, often referred to as the “surge layer” or the “acquisition distribution layer” (ADL; Liu et al., 2019). As currently manufactured, this layer is typically placed between the top skin-facing layer and the absorbent core, and assists the movement of the liquid away from the user’s skin and into the absorbent core (Helmes et al., 2014; Kakonke et al., 2019; Liu et al., 2019). While the ADL accelerates downward wicking, it wicks little horizontally. This leaves sections of the absorbent core saturated and other large areas unused (such as the anterior and posterior sections). An increase in horizontal wicking capabilities would greatly improve user comfort and product efficiency.

Many studies have attempted to improve the permeability, core absorption, and wicking performance of the surge layer. A study done by McDonald (2006) showed that multiply mediums exhibit improved wicking over single ply mediums. It has also been shown that increasing the number of fabric layers in a diaper design increases wicking performance (Herron, 2018). The work presented in this paper further explores the wicking benefits of using an origami-inspired ADL.

### 2.4. Ease of putting diaper on

As previously mentioned, the compression from packaging diapers makes them more difficult to put on. In addition, compressed diapers may not conform easily to the body once worn, which may result in discomfort to the user. While literature does not discuss specific behaviors surrounding adult diaper use, it has been reported that users mitigate these problems by removing the diaper from the packaging and massaging it as a means to “fluff” or “air out” the diaper. Furthermore, users open the compressed diaper and crease it along the long axis in an attempt to reshape the diaper to better fit between their legs. These reported habits suggest that the diaper could benefit from design and packaging features that address these problems.

While individuals of all ages may experience incontinence, a large percentage of users are elderly (Nitti, 2001), and often have limited mobility and poor balance. This makes it difficult for them to lift their legs one at a time. This is made even more difficult when the diaper is compressed and nonconforming. Enhanced diaper shaping would improve these individuals’ ability to use needed incontinence products.

### 2.5. Origami-inspired design

Origami, the ancient art of paper folding, involves a series of folds made into a single sheet of paper. Often this has done to change the paper’s folded appearance into something recognizable, such as an animal or object. A well-known example of this is the traditional paper crane.

Recent advancements in mathematics and engineering have produced methods to incorporate principles of origami art into engineering applications. Putting folds into a material not only changes its appearance, but it can change the overall behavior. When specifically chosen and engineered, origami fold patterns can be used to tune a material’s behavior to enhance a design.

Origami-based design provides a variety of benefits to manufactured products in general. These include: stowability, deployability, part-count reduction, monolithic manufacturability, and reduced assembly (Francis et al., 2014). A subset of origami-based design is origami-inspired design, which is defined as a design that does not directly link itself to origami, but uses certain aspects of origami like folding or geometric shapes (Morgan et al., 2016). Further work has also identified origami-inspired design as using crease patterns, origami-based folding and shape-changing schemes, or aesthetics of origami (Morris et al., 2016). Examples of this category include the James Webb Space Telescope, and the Tessel backpack (Francis et al., 2014). While origami is traditionally a combination of folds along straight lines, folding patterns can also be made from folds along curved lines. A classic example of this is the circular hypar which approximates a hyperbolic paraboloid (Demaine et al., 2011). Curved folding can result in curvilinear geometries and doubly curved shapes. This can be especially useful when designing to mimic the shape of the human body.

## 3. Subsystem Design

### 3.1. Subsystem breakdown

**Figure 1.**
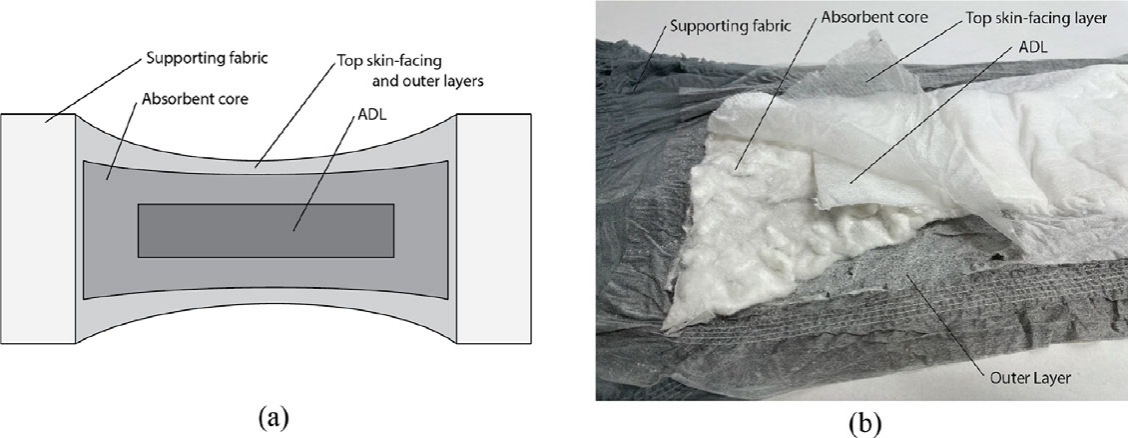
(a) Diagram and (b) photograph showing the components of a typical diaper system.

Traditional diapers contain the following basic subsystems, each shown in Figure 1:

1. *Top skin-facing layer*—Permeable layer that pulls and keeps moisture away from user’s skin.
2. *Acquisition distribution layer—*Assists the motion of the liquid by wicking it toward the absorbent core.
3. *Absorbent core*—Generally made of SAP and wood pulp, this layer absorbs and stores the liquid.
4. *Outer layer*—Nonpermeable layer purposed with keeping moisture inside the diaper.
5. *Supporting fabric*—Holds diaper pad onto the users body.

In this paper, we introduce new diaper technologies for the absorbent core, ADL, and supporting fabric subsystems. Each of these enhancements are described below with their corresponding performance testing and results.

### 3.2. Self-shaping absorbent core

**Figure 2.**
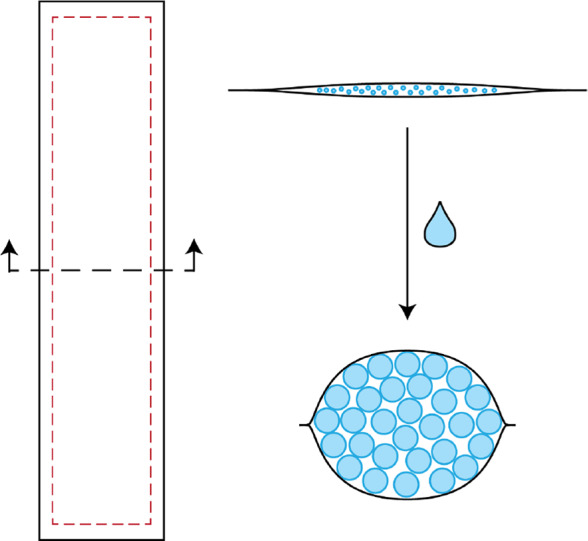
Top view diagram showing the overall shape and placement of stitching in a single tubule. Sew lines are shown in dotted red lines. Cross sections of both dry and wet tubules are shown on the top and bottom-right, respectively. Superabsorbent polymers (SAP) is represented as blue circles between the two membrane layers.

**Figure 3.**
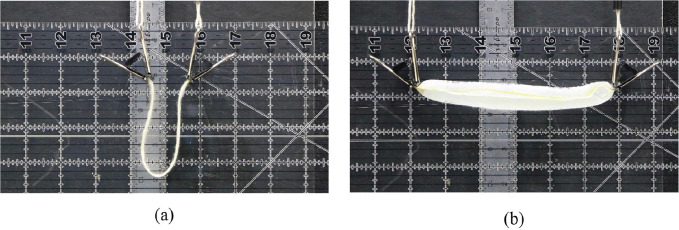
Tubule stiffening test for a single rectangular tubule. Tubule is shown suspended from its ends when (a) dry and (b) liquid activated. The expansion of the superabsorbent polymers (SAP) inside the tubule increases its stiffness and the tubule moves upwards against gravity.

Preliminary testing was done using a laminate composed of two permeable, inextensible membranes adhered at the edges to form a pocket within which SAP was placed. When placed in water, the SAP in the rectangular pocket, or tubule, began to swell as it absorbed the water. A diagram of a single tubule is shown in Figure 2.The swelling of the SAP inside the pocket applied a pressure to the membrane, producing an elongated cylindrical shape from otherwise flat laminate layers. Both dry and liquid activated tubules were suspended from their ends to test changes in stiffness. Figure 3 shows the resulting increase in stiffness for the saturated tubule. This stiffening behavior may be utilized to mitigate bucking and reduce the amount a tubule sags under gravity.

The stiffness observed in the saturated tubule is a result of stress-stiffening (Howell, 2001). Internal pressure induces tensile stress along the length of the tubule which creates resistance to deflection and tube wall buckling.

**Figure 4.**
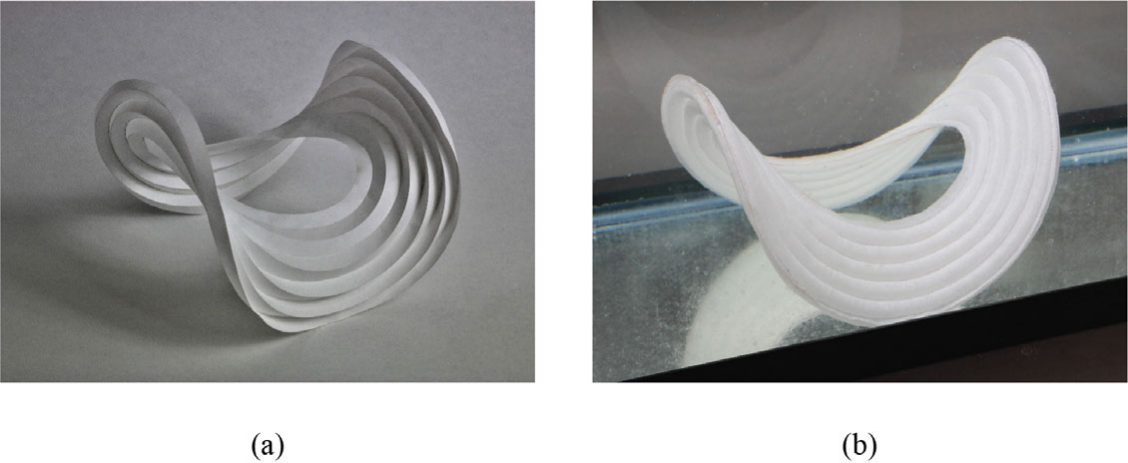
Circular hypar shape made from (a) alternating mountain and valley folds in paper and (b) liquid activation of inextensible membrane.

**Figure 5.**
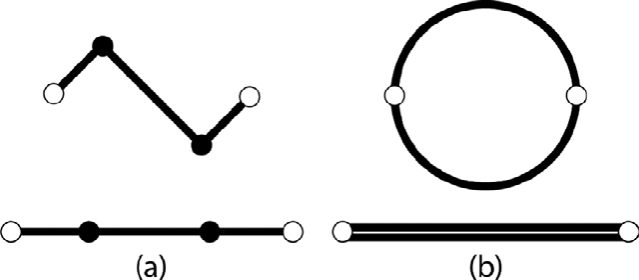
Motion of a (a) folded origami pattern from flat to fully folded state and (b) liquid activated tubule from flat to fully expanded. White circles indicate outermost points and black indicate folds in origami pattern.

Curve-folded origami can be used to create out-of-plane shapes (Kilian et al., 2008; Koschitz et al., 2008; Rabinovich et al., 2019). Research has been done to demonstrate and explain how origami fold patterns can create doubly curved, saddle-like shapes (Demaine et al., 1999, 2011; Dudte et al., 2016). For example, a concentric circular tessellation pattern folded from a flat circular sheet by alternating mountain and valley folds results in a hypar-like shape, as shown in Figure 4a. A similar behavior is observed in a liquid-activated concentric circular tubule pattern, as shown in Figure 4b. This liquid-activated pattern begins as a flat pattern of tubules (as explained in Figure 2), and moved toward the hypar-like shape as the SAP inside the tubules swells. As motion progresses in each of these patterns (flat to folded), the outermost points of each folded unit (shown as white circles in Figure 5) move closer together. The convergence of these points forces the overall pattern into a different out-of-plane shape. The out-of-plane expansion gives both patterns additional stiffness. This shows that the same motion that increases stiffness in the pattern can be used as a method of shaping the membrane.

The stiffness and shaping properties in the liquid-activated tubule patterns shown above demonstrate the feasibility of making out-of-plane, doubly curved shapes. Using concentric tubules, various shapes may be achieved by varying overall pattern geometry. These techniques were used to design an absorbent diaper core with specific purposes to (a) reduce sag and (b) exhibit an improved shape conformance to the body. More specifically, concentric curves can be used to approach the doubly curved shape of the body, while longer straight sections may be used to pass between an individual’s legs. These together both reduce sag and improve shape conformance to the body. With these concepts, combined with experimentation based on experience with hypar folding and prototyping, an absorbent core tubule pattern was designed for use in an adult diaper.

**Figure 6.**
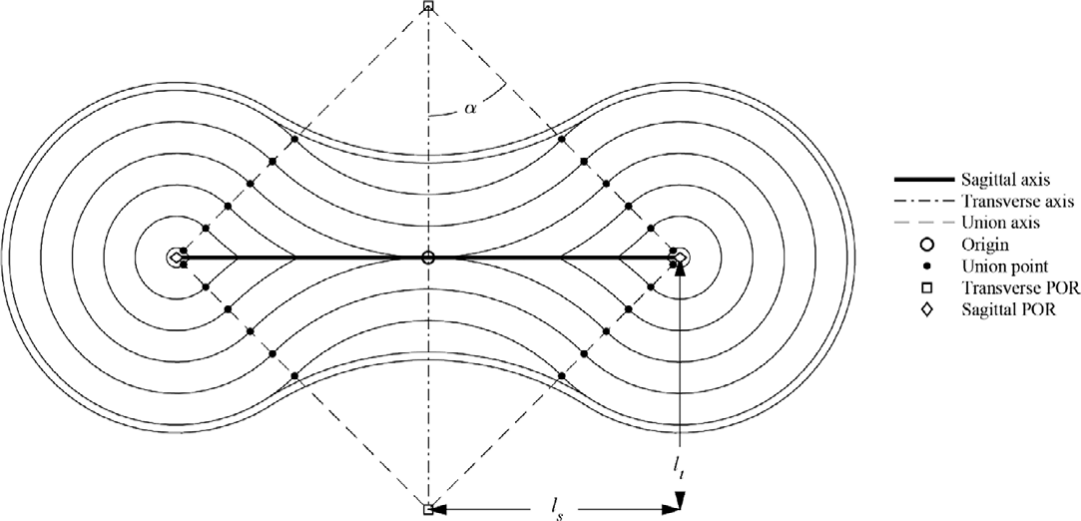
Generalized diagram of tangent four-point radiation (T4PR); 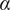 , 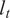 , and 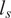 are the union angle, transverse length, and sagittal length, respectively.

The novel absorbent core is a laminate consisting of two inextensible permeable membranes sandwiching a layer of SAP. The two membranes are joined together in a pattern called tangent four-point radiation (T4PR) shown in Figure 6. In the design of T4PR, there exists a sagittal and a transverse axis (thus named to align with the sagittal and transverse axes of the human body). These axes are perpendicular to each other and their midpoints are coincident. The end of each axis is a point of radiation (POR), and there are therefore two sagittal and two transverse POR’s. Half the length of the sagittal axis is termed the sagittal length, and half the length of the transverse axis is termed the transverse length. The sagittal and transverse lengths form a right triangle which is the basis for the geometry of the pattern. Arcs radiate from each POR and constitute boundary constraints between the membranes of the laminate (sewlines in the absorbent article herein). The arcs are constrained to be tangent to each other, as shown in Figure 6. Offsets between sets of tangent arcs can be set to be any width and do not have to be consistent throughout the absorbent. Smaller offsets means more discretion and comfort, however, it also means less liquid absorption capacity. For the prototypes in this paper, the offset between each set of arcs was approximately 12 mm as a balance between discretion, comfort, and absorption performance.

**Figure 7.**
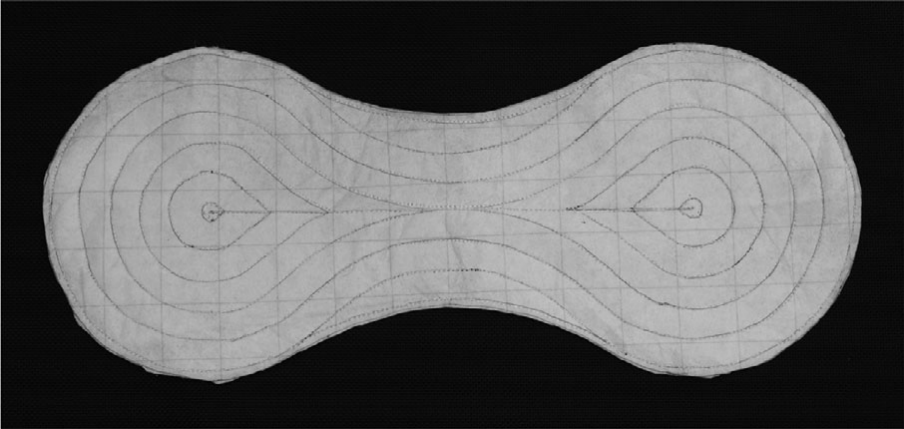
Prototyped absorbent core before liquid activation. Sew lines can be seen in a four-point radiation (T4PR) pattern.

T4PR prototypes were made for shaping and sag testing, and to be tested in a fully integrated diaper. Permeable, inextensible fabric (normally used as stabilizer for embroidery sewing) was used in the prototypes. Prototypes were assembled by lightly spraying one side of the fabric with spray adhesive, followed by a heavy dusting of SAP. The purpose of the spray adhesive was to hold the distributed SAP in place during fabrication. The second layer of fabric was then placed on top, sandwiching the SAP between the two fabric layers. The newly assembled laminate was then joined by sewing along pattern lines using standard cotton thread. A finished absorbent core prototype can be seen in Figure 7.

Diapers require some form of SAP distribution, otherwise SAP will accumulate and result in severe bulging. In a traditional diaper, a wood pulp/SAP matrix enables an even distribution of SAP. In most absorbent core designs, wood pulp accounts for the majority of the thickness and thus increases visibility and decreases discretion. The T4PR design eliminates wood pulp by distributing SAP using spray adhesive and sewlines.

**Figure 8.**
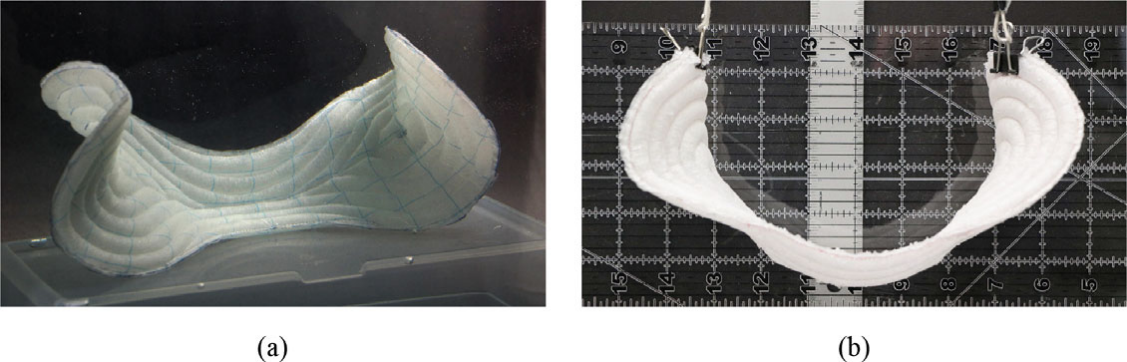
Prototyped absorbent core shown (a) submerged in water and (b) suspended in air from its ends. It can be seen that once liquid activated, the absorbent core takes a designed shape, and resists sag when suspended.

When the prototyped absorbent core is placed in water, the curved tubules expand and transforms the core from its initially planar state into the doubly curved out-of-plane shape shown in Figure 8a. After saturation, the absorbent core was removed from the water and suspended at its ends, as shown in Figure 8b. Resting at its natural shape while suspended, the absorbent core shows a structured shape that resists sag. The doubly curved shape of the sagittal extremes allows the absorbent core to conform upward to the front and backsides of the body while making room for the user’s legs. It can also be seen that the center portion of the activated core forms a stiff trough section. These straight tubules resist sag while also forming a trough that allows the core to better fit a user and guide urine flow away from the sides of the diaper core to prevent leaking.

As a traditional absorbent core absorbs increasing amounts of liquid, it sags downwards from the weight of the liquid. In contrast, as the liquid-activated absorbent core absorbs more water, it becomes stiffer and moves against gravity to take its natural form. This shows that the T4PR-based absorbent core shows promise for conforming better to the body, and also resisting sag.

### 3.3. Pleated ADL

**Figure 9.**
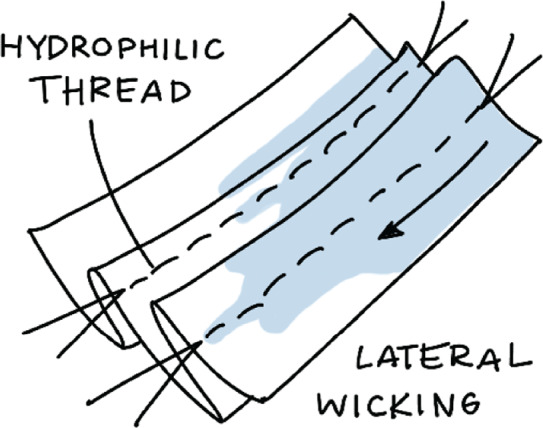
Example of a typical wicking distribution of fluid in a knife pleated material. Note that the liquid wicks furthest along the pleats and threads.

An ADL or “wicking” layer was designed to quickly and evenly distribute fluid throughout the diaper pad. This layer induces increased horizontal capillary action in the wicking material through the introduction of pleats, as illustrated in Figure 9. The resulting fluid distribution carries the fluid to all parts of the absorbent core, thus utilizing a greater percentage of the diaper’s total absorbance capacity and helps the user feel dry.

For wicking and integrated diaper testing, many materials were taken from various brands of diapers for performance testing and prototyping. For the purposes of this paper, each brand of diaper will be assigned and hereafter denoted by a random letter designation in the range A–I. Diapers selected for comparison are: Depend®, Willow, Abena®, Tena®, Because, Tranquility®, Always Discreet®, and Northshore®. Nonwoven material produced by Kimberly Clark—here referred to as “nonwoven stock”—was also used in performance testing. Not every diaper brand was used in every test, as will be noted in each section.

#### 3.3.1. Vertical pleating tests

**Figure 10.**
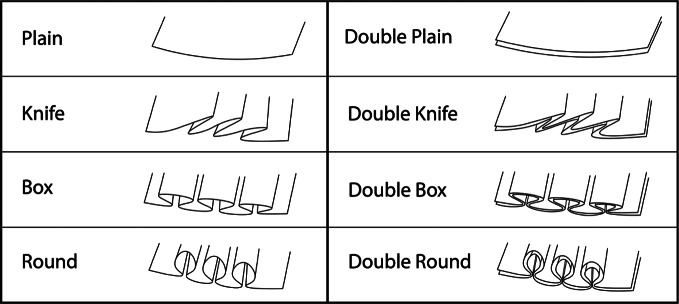
Pleat patterns used in the vertical wicking test. Patterns designated by “double” are identical to their one-ply counterparts but are two-ply.

A vertically oriented wicking test was performed to observe the effect of various pleat patterns on wicking performance. Four pleat types were tested: no pleats (control), knife pleat, box pleat, and round pleat. Each of these pleats were tested using both a single layer and a double layer of material, yielding a total of eight different pleat patterns. An illustration of these patterns is shown in Figure 10.

One test sample of each pleat pattern was made using a jersey mesh athletic wicking fabric. Each had final folded dimensions of 40 × 150 mm. Standard cotton sewing thread was used to stitch the patterns in place. Preliminary testing with the cotton thread demonstrated no significant change in wicking performance.

**Figure 11.**
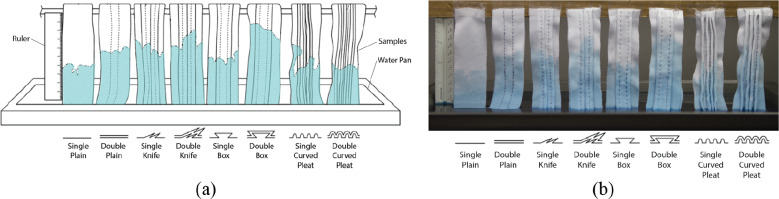
(a) Diagram of vertical wicking test setup. Each type of pleat is represented and labeled. (b) Photograph of actual test subjects. Blue-dyed liquid can be seen on test subjects, wicking vertically.

The testing setup consisted of a shallow container and a horizontal rod elevated 150 mm above it, from which the samples hung. An illustration of this setup is shown in Figure 11. The container was filled with tap water, which was dyed blue with food coloring. The samples were hung from the horizontal rod and lowered into the water simultaneously. During testing, wicking heights were measured every 20 s for a total duration of 3 min. This procedure was repeated three times to verify the consistency of the patterns’ performance.

##### Vertical pleating test results

**Figure 12.**
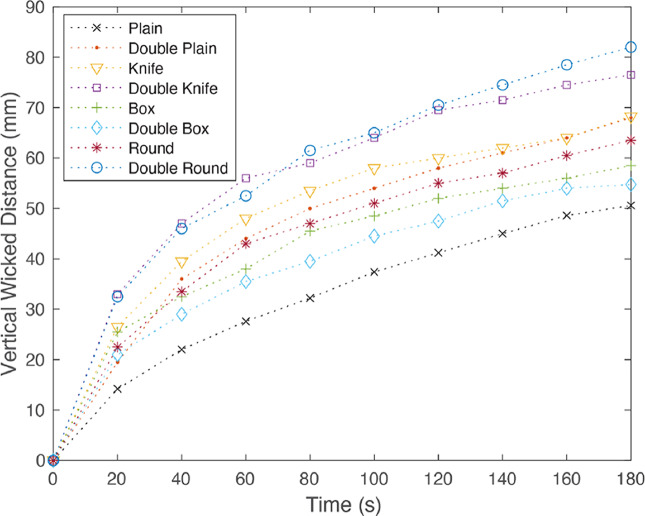
Vertical wicking performance results for each pleat design. Dotted lines are used to show general trends of each pleat design.

Results for the vertical wicking tests are shown in Figure 12. These results indicate that specific pleating patterns show better wicking performance than others, however it can be seen that pleating of any type improved wicking performance in this fabric.

It can be observed that the double round and double knife pleat show the best performance with a wicking improvement of 62 and 51%, respectively. However, these patterns are more difficult to manufacture and require twice the amount of material. On the other hand, the knife pleat is among the simplest pleats to manufacture and still shows a max wicking improvement of 35%. With a similar performance to the single knife pleat, the double plain would also be very simple to manufacture. However, although the double plain pattern had a similar final wicking distance to the knife pleat, it exhibited a slower initial wicking performance. Considering ease of manufacture, final and initial wicking performances, the knife pleat pattern was chosen for further analysis.

It should be noted that the fabric used was an absorbent option used to show the relative effectiveness of different pleat styles and not the absolute wicking distances we would expect to see in more specialized fabric.

#### 3.3.2. Horizontal testing

**Figure 13.**
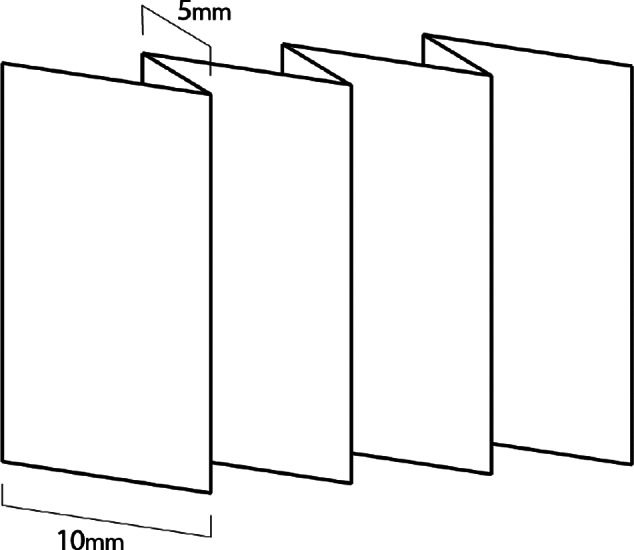
Final pleated surge layer pattern and dimensions, shown in its folded state. It includes a knife pleat along its long axis to improve wicking performance.

Tests were performed to examine the effects of pleating on current commercial ADL performance in the horizontal plane. Samples from brands A, B, D, F, G, H, and I, were used in these performance tests. Straight knife pleats of 10 mm, spaced 5 mm apart, were folded and sewn into one of each surge layer brand, as shown in Figure 13. Each newly pleated ADL and unpleated ADL’s were tested to compare wicking performance.

Each sample was suspended horizontally between two vertical supports at a freestanding length of 155 mm with neutral tension. Synthetic urine was used in these tests, made using urea 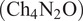 , sodium chloride ( 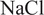 ), potassium chloride ( 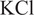 ), and sodium phosphate 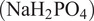 dissolved into distilled water (Shmaefsky, 1990). Blue dye was added to the mixture to aid visibility of the results. A portioned amount of synthetic urine was delivered to the center of each material sample using a large syringe with a silicone tube extension. A volume of 110 ml of synthetic urine was transferred to the sample at a rate of 11 ml/s for each test. This insult quantity is one-third the average void quantity of continent senior males and females (Kumar et al., 2009) and was used as an approximation of an incontinent senior population.

The total wicking distance was measured using two different criteria to capture the overall wicking ability of each sample. Distances past 155 mm were not recorded as this would mean the urine has met the edges of the test setup. The maximum wicking distance was measured as the largest longitudinal distance between points of liquid in a sample. However, it was observed that while a small amount of liquid can wick fairly far, particularly along thread and pleat lines, the greater part of the liquid wicks a much shorter distance. An illustration of this is shown in Figure 9. For this reason, a “bulk” measurement was also taken to record the distance to which the majority of the liquid was able to wick. Bulk distance was defined as the longitudinal length of the main mass of the liquid, excluding any narrow stretches of liquid wicked along thread or pleat lines. Both of these distances (bulk and max) were measured through use of a ruler placed parallel to the material samples. Photos of the results were also taken for further post-test analysis.

**Figure 14.**
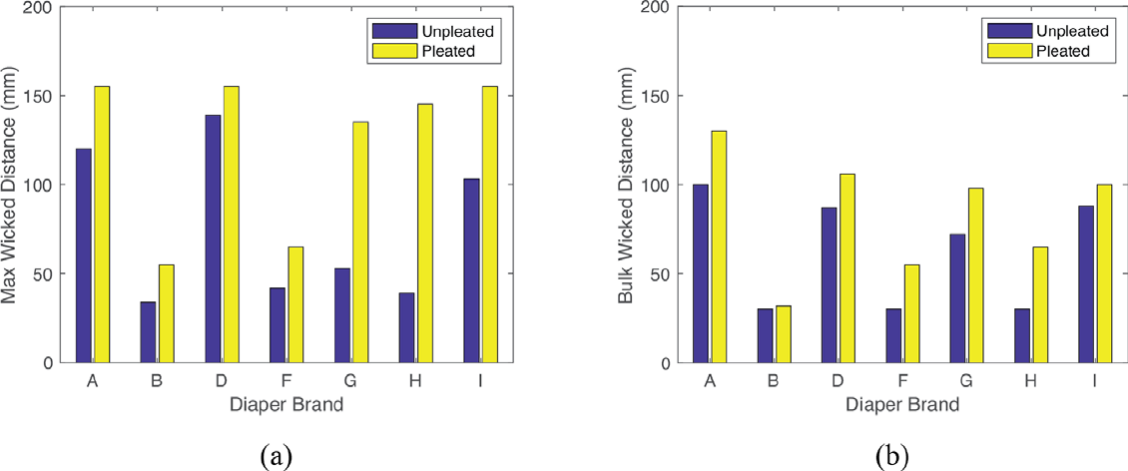
Wicking results for baseline and pleated testing reported in (a) max wicking distance and (b) bulk wicking distance.

Wicking results are shown in Figure 14. Similar to the findings of the vertical wicking test results, we can see that any pleating in the material improved horizontal wicking performance. It can be seen that the pleated materials from brands A, D, and I all wicked a max distance of 155 mm. Pleats in the brand I material increased the bulk wicking distance by 14% and maximum distance by 50% before achieving the limits of the experimental setup.

In summary, incorporating knife pleats into an ADL increases wicking performance.

#### 3.3.3. Pleated surge layer design

Using the results from the above tests, a wicking layer was designed which consisted of an ADL with knife pleats of 10 mm spaced 5 mm apart. This basic fold pattern and dimensions of the final wicking layer design are shown in Figure 13.

**Figure 15.**
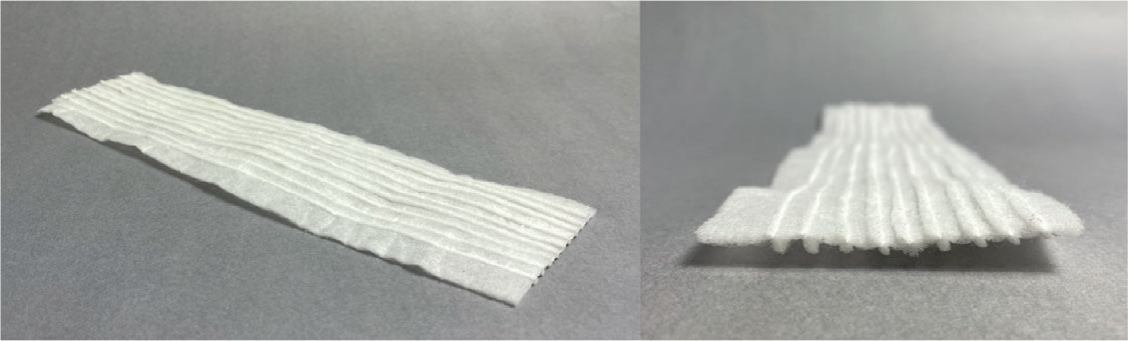
Final pleated acquisition distribution layer (ADL) prototype.

A prototype was made for use in integrated diaper testing. The prototyped wicking layer was made from an ADL material taken from Depend®brand, with nine knife pleats sewn into it along its long axis. The final prototype is shown in Figure 15.

### 3.4. Deployable waistband

A disposable waistband was designed as a means to deploy the diaper once it was removed from its packaging. This deployed diaper waistband would make it easier for users to put the diaper on. When packaged, the waistband is folded and compressed with the diaper; once the packaging is removed, the stored strain energy in the waistband deploys, thereby opening the diaper. While solutions to open the diaper could include a simple solid band (such as a tape-measure structure with foldable joints) placed into the waistband, it would not stretch with the fabric. This stretching motion is necessary as it allows an individual to put the diaper on. A waistband system is needed that folds with the diaper when packaged, deploys the diaper when opened, and stretches with the diaper waistband to allow dressing.

#### 3.4.1. Lamina emergent torsion joints

Lamina emergent torsion (LET) joints (Jacobsen et al., 2009) and several LET variants (Wilding et al., 2012; Delimont et al., 2015; Xie et al., 2017; Xie et al., 2018a; Xie et al., 2018b; Chen et al., 2018; Qiu et al., 2021) have been used in many engineering applications ranging from space applications (Hamza et al., 2019; Hamza et al., 2020; Hwang et al., 2020; Pehrson et al., 2020) to spinal implants (Stratton et al., 2010). These surrogate folds can be used to replace a standard hinge by cutting designed geometries into adjacent panels. When the LET joint is actuated in a hinge-like motion, the members are torsionally deflected and strain energy is stored. When released, the stored strain energy in the LET joint moves the panels back to their low-energy states.

While the geometry of a LET joint allows a hinge-like motion, it also introduces other undesired motions, referred to as parasitic motions. LET joints can be designed to be flexible in bending and stiff in other degrees of freedom. Such a design replicates hinge-like behavior. While often undesirable in most applications, the parasitic motion observed with a compressive or tensile load could be utilized when elongation along that axis is desired. Pehrson et al. (2020) presented a way to design for motion along three degrees of freedom in a LET joint including tension/compression, deflection from in-plane moments, and the desired hinge-like motion.

#### 3.4.2. Proposed diaper waistband

**Figure 16.**
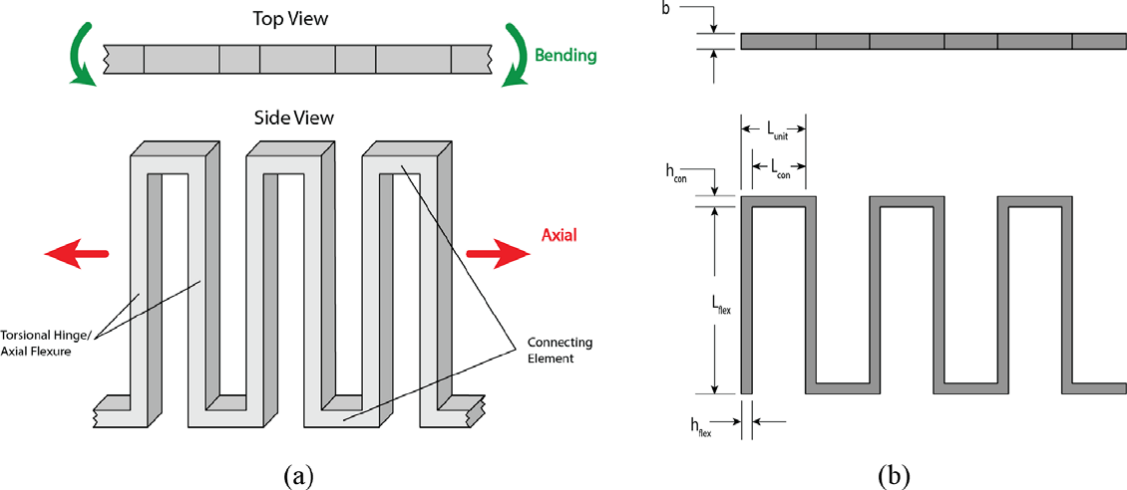
(a) Basic elements of a lamina emergent torsion (LET) Band unit. Displayed are three of the LET Band units that make up the waistband. (b) General dimensions defined for a LET Band unit. 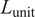 defines the length of each unit while 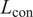 defines the connecting element length.

**Figure 17.**
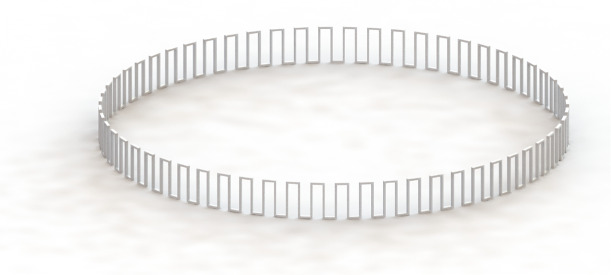
Completed Lamina emergent torsion (LET) Band system with a circumference of 28 inches and dimensions reported in Table 1.

A modified LET joint was designed into a plastic waistband as a means to deploy and structure an adult diaper. Similar to the standard LET joint, it is made up of long flexures that can deflect in both torsion and bending. The basic framework of the “LET Band” system is shown in Figure 16a and the complete waistband with repeated units is shown in Figure 17.

The torsional hinges in a standard LET joint are designed to be flexible in torsion and stiff in bending. This makes the joint as a whole flexible in bending and stiff in tension. This allows the desired hinge-like motion while limiting the amount of parasitic motion in the joint. In contrast, for use in a diaper, it is required that the waistband stretch with the fabric, and deploy/hold the diaper into an open shape. The fabric-like stretch behavior can be achieved by making the diaper waistband flexible in axial tension, requiring the individual flexures in the LET Band to be flexible in bending. The deployable behavior can be achieved by making the diaper waistband stiff in bending (to resist folding in on itself), requiring the individual flexures in the LET Band to be stiff in torsion. As a note, these are opposite to the desired characteristics in a standard LET joint flexure.

Dimensions of the LET Band units can be calculated to maximize the flexure torsional stiffness and minimize the bending stiffness. As shown in Budynas et al. (2005) and Chen and Howell (2018), the torsional stiffness of the rectangular cross sectioned flexure can be calculated by.

The torsional stiffness, 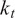 , can be defined by
(1)

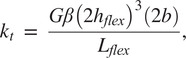

where 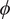 is the angle of twist, 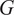 is the Shear Modulus, 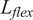 is defined as the flexure length, 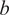 is defined as the flexure thickness, 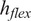 is the flexure width (see Figure 16b), and 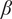 is a function of the ratio 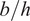 which increases to 0.333 as 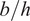 approaches infinity (Timoshenko, 1940). A closed-form expression for 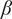 can be found in Chen and Howell (2009).

As shown by Howell (2001), the bending stiffness, 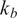 of a rectangular flexure can be calculated by
(2)

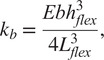

 where 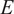 is Young’s Modulus and 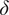 is deflection.

To maximize the benefits of the design, as explained above, the flexures must maximize the torsional stiffness and minimize the bending stiffness. In other words, minimize the ratio
(3)

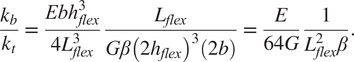



Using equation (3), we can see that increasing 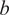 and 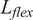 , while minimizing 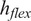 will give us the most favorable design for these requirements.

It is important to note that there is a range of feasible values that can be used in the application of an adult diaper waistband. In other words, increasing *b* and 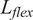 improves the desired force performance, however, the more we increase *b* and 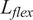 the more uncomfortable it would become to the user. Therefore, we must define upper bounds for *b* and 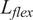 that would ensure a comfortable waistband. For our prototypes and testing, a waistband with 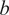 = 1.5 mm and 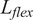 = 17.5 mm were used as those are approximately the corresponding dimensions of a typical leather belt. Dimensional constraints may be changed for desired performance.

After 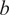 and 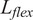 are defined, connector length ( 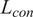 ) can be defined using the desired stress in the flexures and the desired axial elongation amount of the waistband. In an adult diaper, the waistband must elongate and increase its circumference to allow the user to put their feet through and pull the waistband over their hips. On a similar note, the undeflected waistband circumference must also be small enough to hold the diaper on the user’s waist. The difference between the undeflected circumference ( 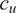 ) and the deflected circumference ( 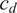 ) two defines how much combined deflection ( 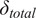 ) the flexures must undergo. Thus
(4)

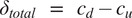

 and the deflection per flexure, 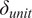 , can be solved using
(5)

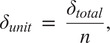

where 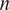 is the number of units. Note that in this waistband design, 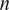 must be even to create a continuous loop. The max stress, 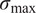 in each flexure can be solved by
(6)

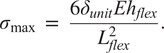



Rearranging we get
(7)

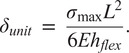



We then can solve for the minimum number of flexures, 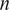 , we need in order to undergo the total deflection without exceeding the max stress.
(8)

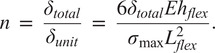



The needed connector length, 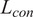 , can be determined to define our geometry.
(9)

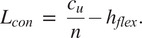



Final dimensions for the LET Band units used in the diaper waistband are reported in Table 1.

**Table 1.** Final dimensions used for prototyping and in diaper integration testing

Parameter	Value (mm)
*L*_*flex*_	17.5
*b*	1.5
*h*_*flex*_	1
*h*_*con*_	1
*L*_*con*_	7
*L*_*unit*_	12

A prototype waistband was 3D printed from PLA material for implementation and testing. Each waistband was printed in four sections that were connected into final form using cyanoacrylate adhesive.

Prototyped waistbands were sewn into the waistband section of the diaper material to encase the PLA prototype. The completed diaper-waistband prototype was then folded up as it would be in packaging, and then released to observe the waistband’s deployment behavior.

#### 3.4.3. Waistband results

**Figure 18.**
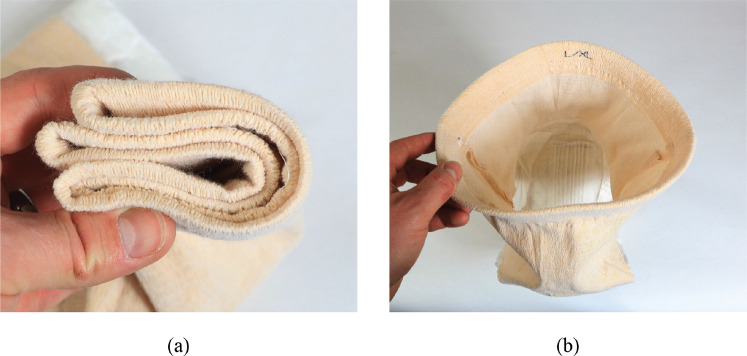
Integrated waistband prototype in (a) folded state and (b) deployed state.

Figure 18 shows the prototyped waistband being used in a full diaper prototype in a folded and deployed state. The waistband allowed the diaper to be folded tightly allowing the diaper to be stored compactly. Once released, the waistband moved towards its lowest energy state, forcing the diaper into its opened position.

Once opened, the LET band system exhibited the same elasticity along the waist as the external fabric. In other words, the LET band is able to stretch along its length with the fabric to allow the user to pull the diaper over their hips and conform to the shape of their waist.

The deployed diaper remained fully open while being supported from one point along the waistband. This suggests that the diaper would remain in its opened shape if the user were to put the diaper on while using only one hand to step into it.

## 4. Integrated Diaper System

**Figure 19.**
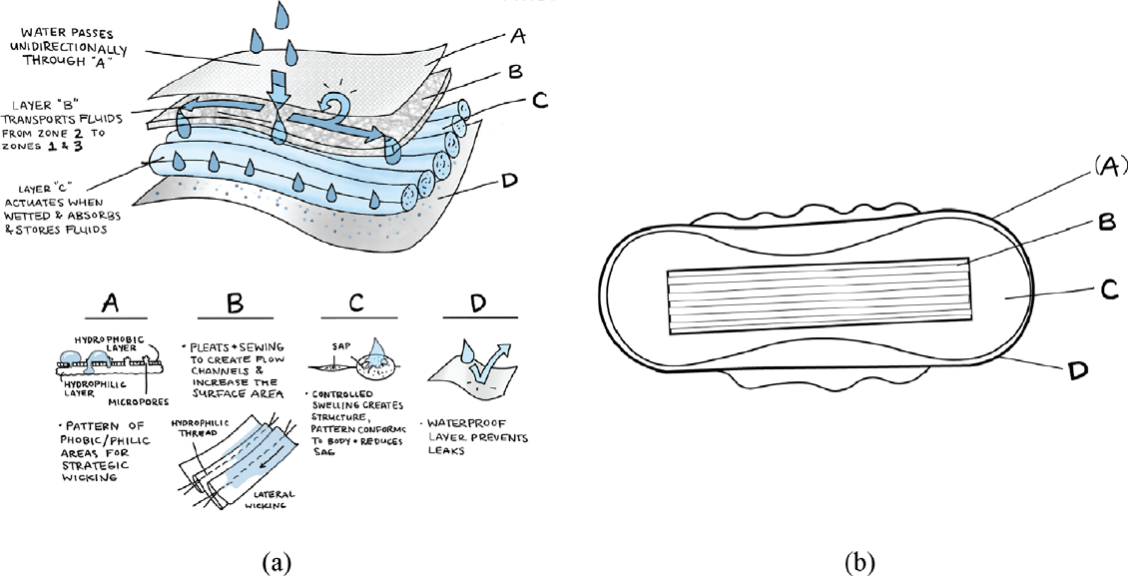
(a) Diagram showing the path of liquid through and interaction between diaper pad layers. (b) Layout of diaper pad layers as seen from above.

**Figure 20.**
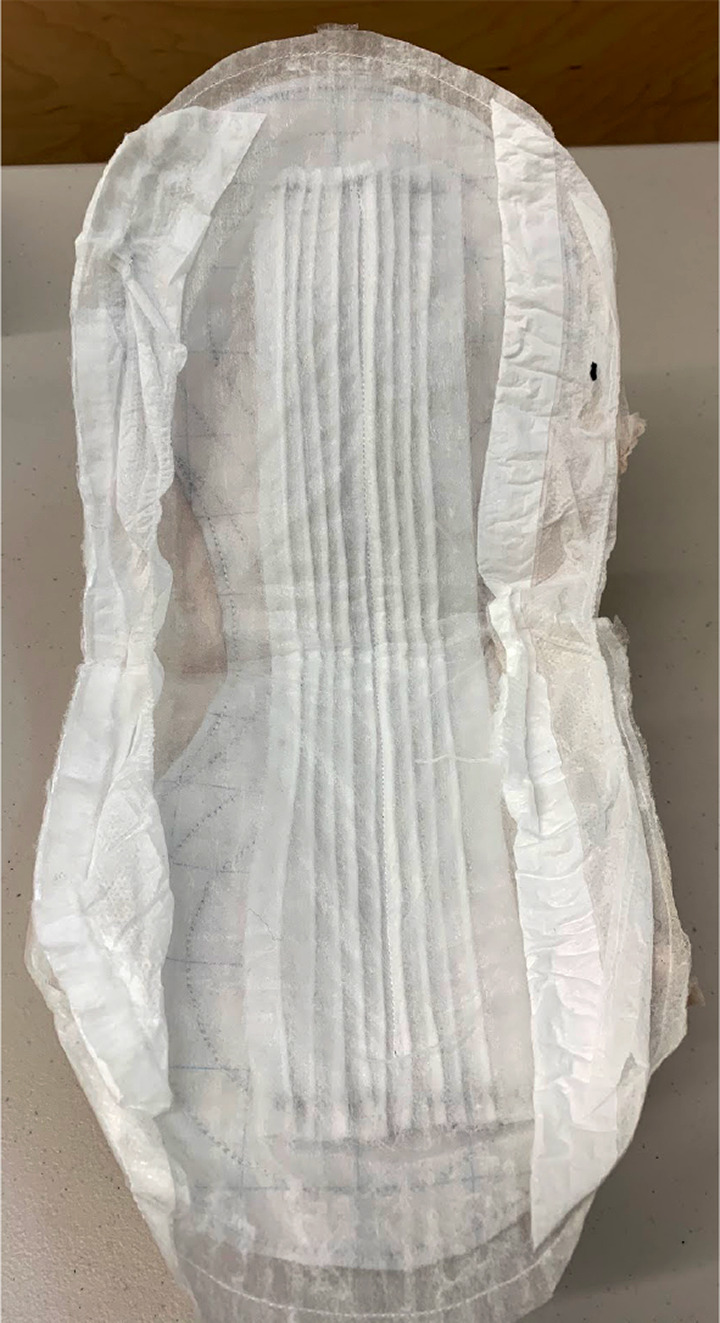
Prototype of integrated diaper pad, later placed into the outer diaper fabric.

Each of the above concept designs were integrated into a full diaper design that could then be prototyped, tested, and compared to current diapers on the market. The final integrated diaper prototype was assembled from a T4PR designed absorbent core, single knife-pleated ADL, and LET Band system insert. Material used in the top skin-facing layer and bottom-most waterproof layer used in prototyping the integrated diaper are made using Northshore brand diapers. The integrated diaper pad was then placed into an external brief-like shaped layer of cloth that is also used in Willow Brand diapers due to its ability to stretch anisotropically. Figure 19 shows the conceptual layout of the layers in the prototyped diaper, including the interactive flow between subsystem layers. Figure 20 shows an integrated pad prototype, including a top skin-facing layer, pleated ADL, absorbent core, an impermeable external layer, and leg gathers. This pad was added to the Willow brand external brief-like layer, and a LET band system was sewn into the waistband.

### 4.1. Testing

To analyze the performance of the finalized diaper, comparison tests were conducted against five other diapers currently in the market: Tena®, Because, Depend®, Willow, and Abena®. These will be referred to diaper brands “A”, “B”, “C”, “D,” and “E”, though not in that order. These diapers were chosen because of their popularity in the market.

#### 4.1.1. Dry discretion testing

**Figure 21.**
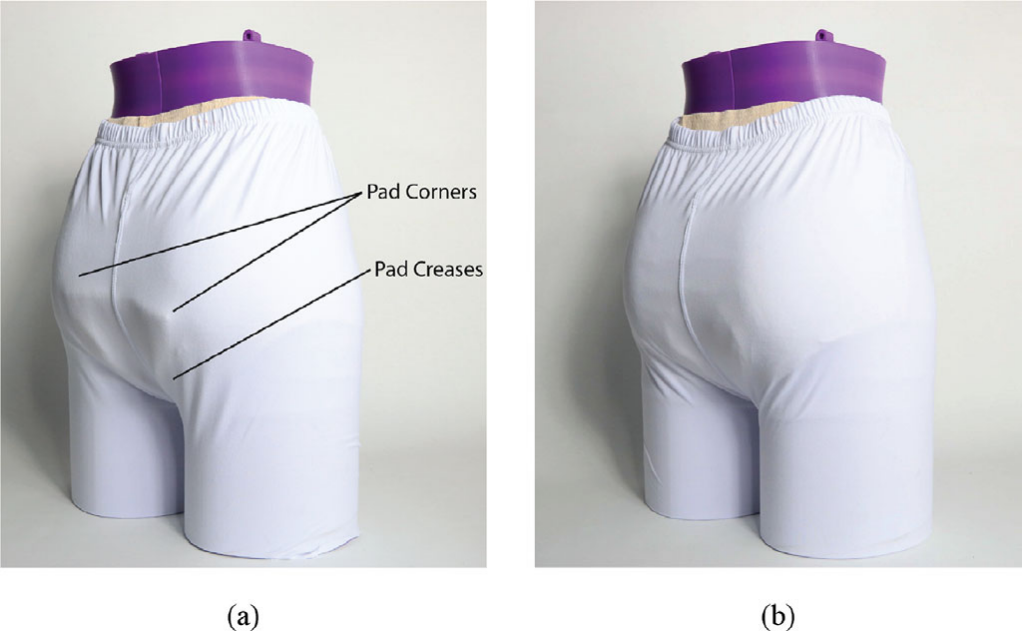
(a) Dry discretion results for Brand A diaper. Common visible creases and bulges such as pad corners and pad creases are shown. Similar results were found in all tested current-market diapers. (b) Dry discretion results for integrated CMR diaper. Improved discretion can be observed through the eliminated pad corners, and thinner absorbent core, making creases less noticeable.

Testing was done to identify the creases and bulging that can be seen while wearing an adult diaper underneath clothing. A dry diaper of each brand was placed on a mannequin, and tight-fitting pants were then placed over the diaper. A visual inspection was done to identify creases and protrusions caused by the diaper that could be seen through the pants. During testing, lighting was used to create high contrast, accentuating the shadows caused by the abnormal diaper contours. The mannequin was photographed at several angles to capture observations. Example images can be seen in Figure 21.

#### 4.1.2. Sag testing

**Figure 22.**
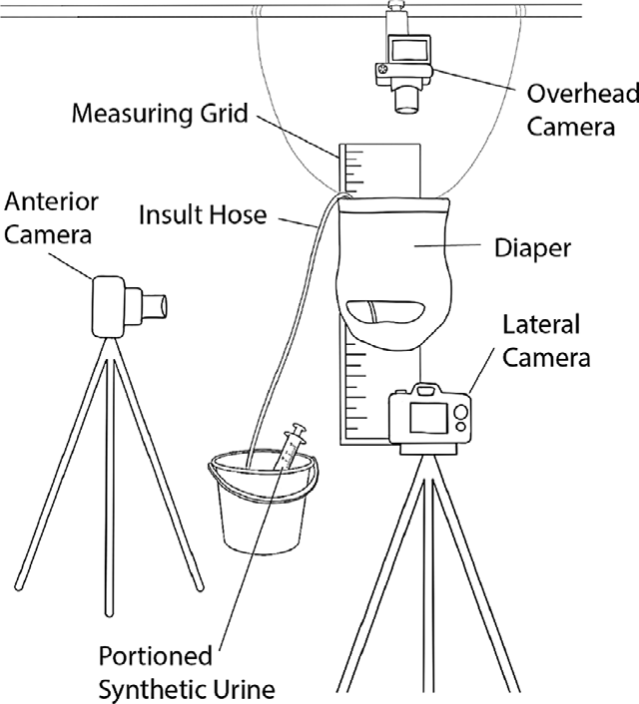
Layout diagram of wicking test setup postinsult.

Sag performance of each diaper was tested using the setup depicted in Figure 22. Each diaper was placed around a metal suspension hoop, hung from above and centered on the backdrop. Cameras were placed from above, front, and side of the stage to capture the needed measurements. Once in place, and datum measurements taken, the diaper was wet in a way to simulate an incontinence episode.

Synthetic urine was again made to simulate the salinity of real urine (Shmaefsky, 1990), and blue die was added for visibility. The synthetic urine formula was the same as that used in the horizontal wicking tests.

A plastic syringe and tubing were used to deliver synthetic urine to the location in the diaper that corresponds with the position of a female urethral orifice. The insult quantity and rate were 110 ml and 6.5 ml/s, respectively. After the liquid was insulted, photographic measurements were taken to record the position of the filled diaper. Sag amounts were calculated through photo analysis with reference to the starting datum of each brand.

Through preliminary testing it was found that as the SAP in an absorbent core grows, it forms a lattice-like structure which helps it sag less. However, once the core is disturbed/moved, the structure is broken up and the sag of the diaper increases. In consumer use, this happens when the user walks or moves once the diaper is wet. Due to these observations, after initial sag measurements were taken, each diaper was agitated by lightly squeezing with the hands several times to simulate walking. Afterwards the diaper was allowed to sag once more and photographic measurements were taken.

#### 4.1.3. Wet discretion testing

After urine was injected into the diapers for sag testing and measurements were taken, each diaper was placed on a mannequin to again identify visible creases and protrusions underneath clothing. The same process used for dry discretion testing was used in this test. A visual inspection was done to identify creases and bulging caused by the wetted diaper as observed through the clothing. The mannequin was photographed at several angles, and notes were made to record observations.

#### 4.1.4. Wicking testing

**Figure 23.**
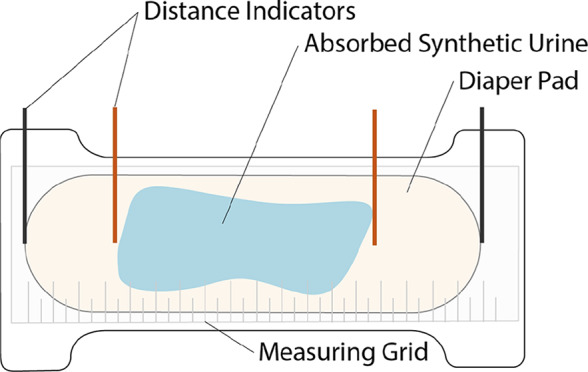
Layout diagram of wicking test setup postinsult.

Total wicking of each diaper was measured after insult. Each diaper was opened and laid flat under a transparent ruler grid. A visual examination was done and the distance to which the synthetic liquid wicked was marked. An example of this setup is shown in Figure 23. Orange markers were used to mark the wicking distance, and the black markers denote the edges of the pad. After markers were placed, each diaper was photographed for later analysis. Area coverage of the blue synthetic urine was made using a rectangular approximation with measurements from orange and black markers.

### 4.2. Results

#### 4.2.1. Dry discretion

Nearly all of the diaper brands showed several creases and bulging visible through the tight-fitting pants. Figure 21a uses diaper brand A as an example to show where these indiscretions were observed, including pad corners and creases. Similar findings were observed in all tested current marketed diaper brands.

Due to their rectangular geometry, pad corners were the most visible, particularly over the buttocks region. This is because the corners—a normally indiscreet shape—are accentuated by their location over the most prominent portion of the buttocks region. In addition, the thickness of the diaper pad contributes to the prominence of the corners. With the exception of brand A diapers, all tested diapers use wood pulp in their absorbent pads to assist in liquid distribution and retention. This wood pulp is bulky, however, and contributes the majority of the pad thickness, making the diaper less discreet. Results showed that all marketed diapers in this test had visually prominent rear pad corners.

The CMR diaper showed improved discretion through the elimination of the pad corners. Figure 21b shows the CMR diaper results, in comparison to Figure 21a. It was observed that the curved geometry of the pad eliminated the prominent protrusion of corners, and more closely resembles shapes exhibited by the body.

In addition to visible pad corners, all diapers showed pad creases near the lower end of the buttocks (see Figure 21b). These were caused by the diaper pad folding in an attempt to fit between the legs. It was observed that the thicker the absorbent core, the more noticeable these creases were. Tests results showed that diaper brands that use wood pulp in their absorbent cores (brands B–E) showed more prominent creases through clothing due to the thicker absorbent core. On the other hand, tested diapers that do not use wood pulp (brand A and the CMR diaper) had less visible pad creases.

In summary, the CMR diaper’s curved pad perimeter eliminates the pad corners, making the diaper more discreet. Furthermore, the CMR diaper does not use wood pulp which makes the absorbent core thinner resulting in less visible pad creasing.

#### 4.2.2. Sag

**Figure 24.**
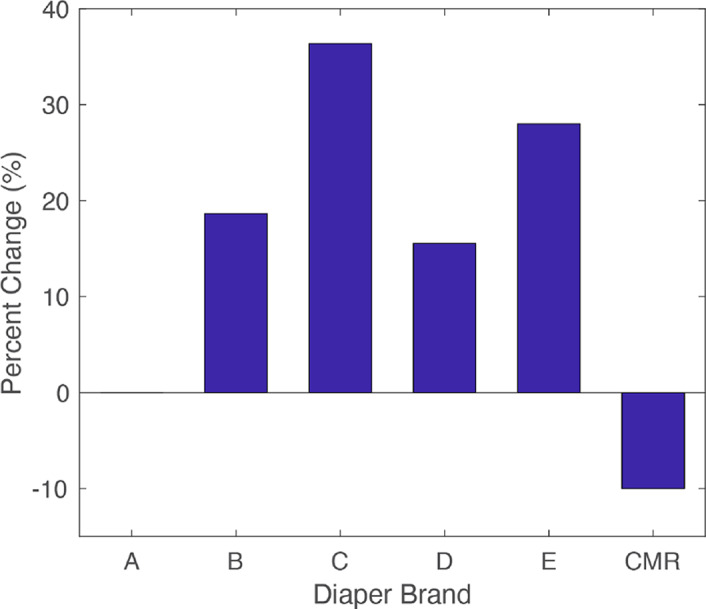
Diaper sag displacements after liquid insult. Shown are sag measurements for each diaper pre- and postagitation.

**Figure 25.**
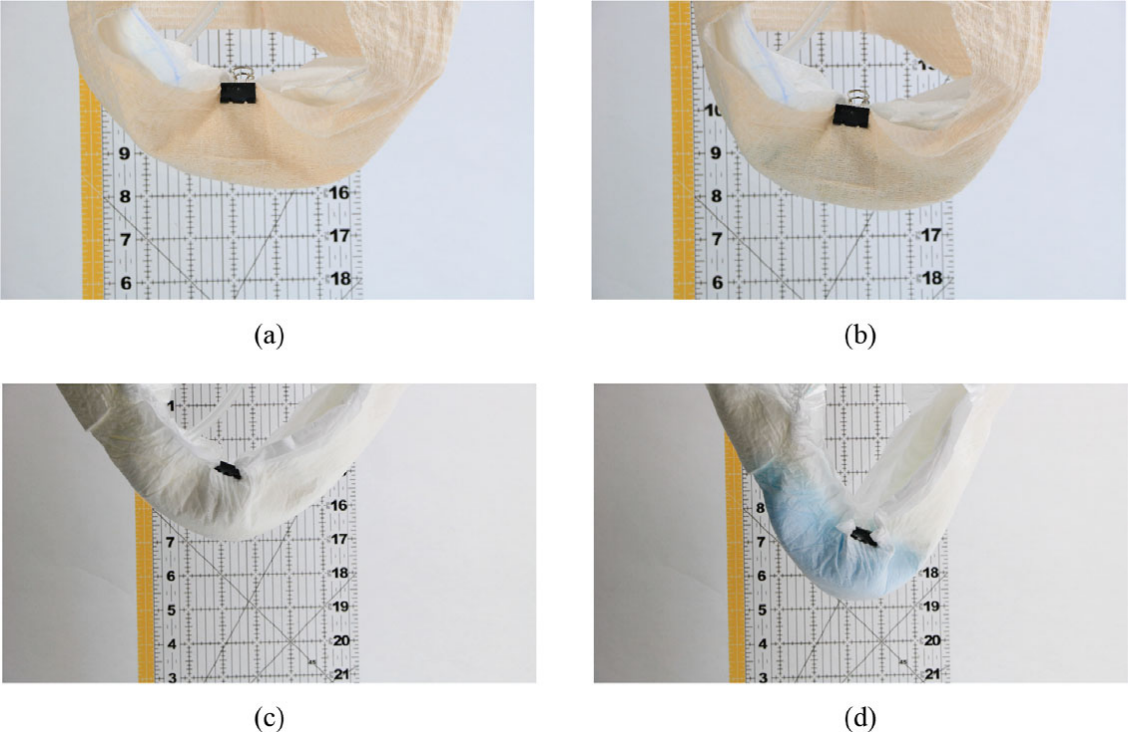
Example before and after insult comparison pictures during sag testing. CMR diaper is shown (a) preinsult and (b) postinsult. The brand B diaper is also shown (c) preinsult and (d) postinsult.

**Figure 26.**
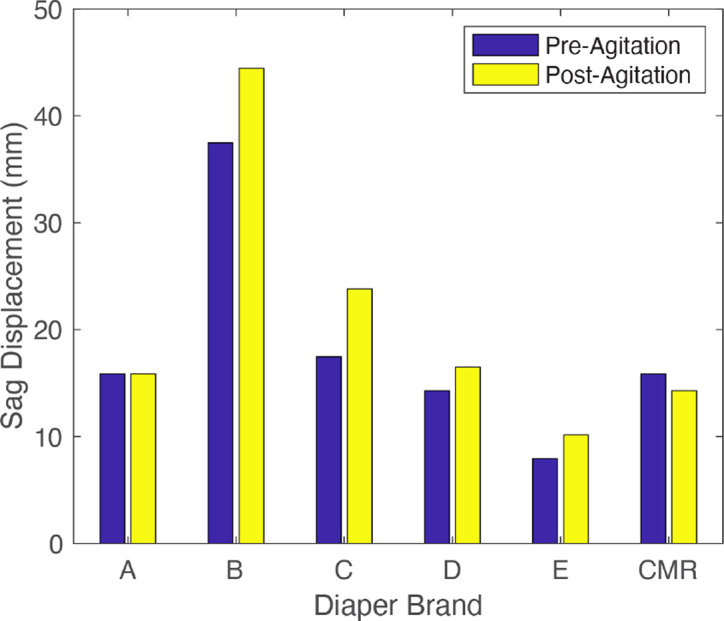
Percent change in measured sag due to diaper agitation.

Figure 24 shows pre- and postagitation sag displacements of each diaper. Figure 25 shows pre- and postinsult positions for the CMR diaper and the brand B diaper. In addition, the percent change of sag displacement between pre- and postagitation was calculated and is shown in Figure 26. It was observed that while all other diaper sag increased after agitation, the CMR diaper decreased in sag. This is further evidence that the CMR absorbent core utilizes a liquid-activated actuation mechanism that causes it to work toward a stiff, predetermined shape, even against gravity.

#### 4.2.3. Wet discretion

**Figure 27.**
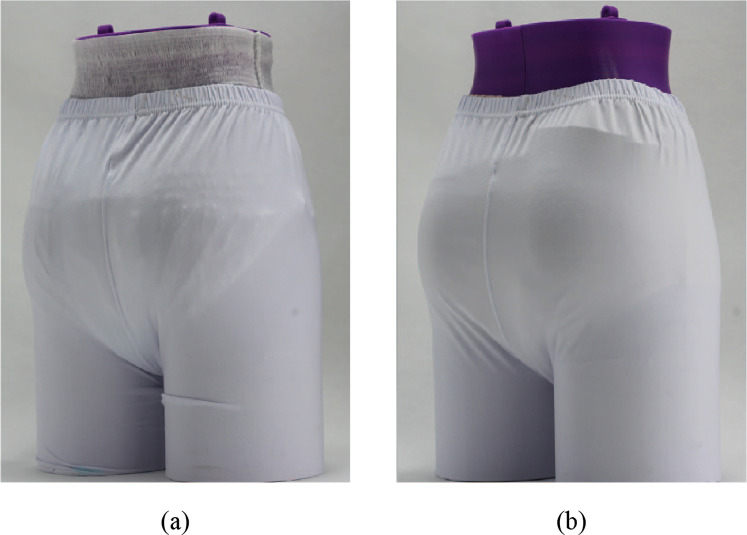
Wet discretion results for (a) brand C diaper and (b) CMR diaper. Pad corners, creases, and sag can be seen in the brand C diaper. Slight sag and creases can be seen in CMR diaper.

Figure 27 shows the final results of the brand B diaper juxtaposed next to the CMR diaper. Results for other diapers were similar to those of the brand B diaper. As seen in the dry discretion testing, pad corners remain visually prominent in diaper brands A–E. In addition, it was observed that bulging, sagging, and pad creasing are visible in all diapers, although attenuated in the CMR diaper. In summary, the CMR diaper improved wet discretion performance through a curved pad design and reduced sag.

#### 4.2.4. Wicking

**Figure 28.**
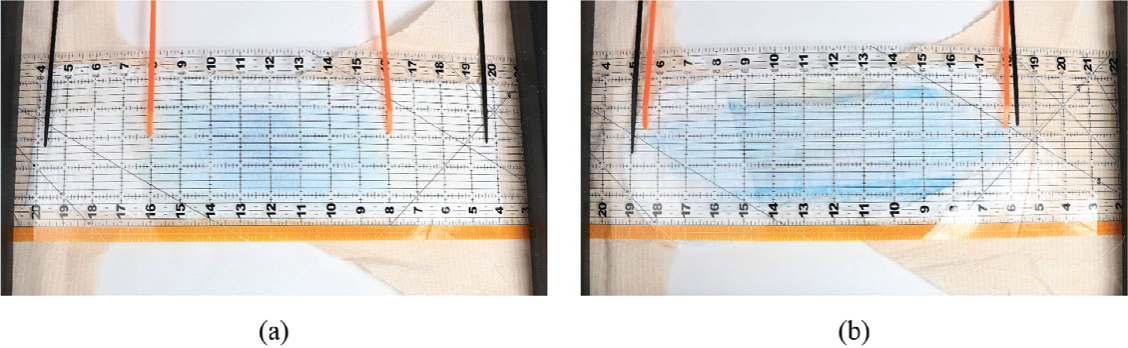
Wicking results for (a) brand A diaper and (b) CMR diaper. Black markers indicate absorbent pad edges, and orange markers indicate furthest distance wicked.

Figure 28 shows integrated wicking results for the brand A diaper and the CMR diaper. Results other diapers were similar to brand A, and are reported in Table 2.

**Table 2.** Measured wicking distances of each diaper brand

Diaper	Wicked length (mm)	Pad coverage (%)
Brand A	203.2	52
Brand B	209.6	52
Brand C	273	56
Brand D	225.4	62
Brand E	187.3	42
CMR	324.3	93

The average wicking distance and pad coverage percentage of the tested current market diaper brands was 219.71 mm and 53%, respectively. The integrated CMR diaper wicked 43% further than current competitors, and utilizes an average of 40% more of its pad. This suggests that the knife pleated surge layer, integrated into a full diaper design improves wicking distance and improves absorbent core efficiency.

It is important to note that the integrated testing was done with only one sample from each brand. This was done as a proof-of-concept demonstration and not a characterization of diaper performance. In addition, while all tested diaper pads were of the same width, they varied in length. This affects the reported pad coverage percentages, but also illustrates that current pad designs include much material that goes unused due to little or no horizontal liquid distribution.

## 5. Discussion on Materials

Many different fabrics were used throughout the prototyping phase of this project. While some limited testing was done to find effective fabrics, fabric options were limited to those that could be stripped and repurposed from other diapers and fabric that could be purchased in small quantities.

### 5.1. Top layer

In the final prototypes, the top layer was taken from Northshore brand diapers and worked efficiently to allow water to pass through it. This brand of top layer was used primarily due to its size which allowed it to be repurposed. The performance of the CMR diaper would be further improved through the implementation of a specialized high-performance fabric designed for diaper top layers.

### 5.2. Absorbent core

For the absorbent core to be effective, the material must be highly water-permeable. This allows the liquid to pass quickly through the fabric, giving the liquid immediate access to the SAP.

For the absorbent core to take the desired shape, it is important that the material not elongate when pulled in any direction. This is because the swell pressure in the tubules give the absorbent core its shaping properties. If the material were to stretch when placed under the swell pressure, the layer would not immediately take the desired shape and the sag performance would be negatively affected.

In final prototypes described in this paper, sheet fabric material that is generally purposed as an embroidery stabilizer was used due to its resistance to plane strain. This material, however, is not designed to be used with liquid and was therefore not optimized for liquid permeability. During testing, it was observed that the material caused liquid to pool on top of the absorbent core before slowly soaking through to the SAP.

Because spray adhesive was used to hold the SAP in place as the two fabric materials were sewn together, it is likely that this adhesive covered a fraction of the SAP and prevented it from absorbing as much and as quickly as it would have without the spray adhesive. A manufacturing process that eliminates the use of spray adhesive would likely improve the liquid absorption rate.

The implementation of a higher-performing material would greatly improve the performance of the absorbent core. This would include resistance to plane strain and high water permeability. A fabric with these properties would allow the liquid would pass to the SAP more quickly and pooling would greatly decrease.

## 6. Conclusion

Current adult diaper technologies work to improve the discretion, wicking, sag, and usability of adult diapers. This paper has proposed many designs that may be implemented to improve diaper performance.

A self-shaping absorbent core was designed and tested. Once exposed to liquid, the absorbent core utilizes stress-stiffening to increase stiffness against sag and to change its overall shape to better fit the body. It has been shown that the T4PR absorbent core design improves shaping and reduces sag.

A pleated acquisition distribution layer design was presented, prototyped, and tested. The knife pleated ADL was shown to improve wicking performance over that of current ADL designs.

To make it easier to put the diaper on, a deployable waistband design, the LET Band system, was proposed. When placed into the waistband of a disposable diaper, the band folded and stretched with the diaper material, was would be observed in normal diaper use. In addition, when the diaper was released from a folded state, the LET band system moved the diaper into an open position. This make it easier for the user to put the diaper on by forcing the diaper into an open position.

All three subsystem designs were integrated into a single diaper system. Through testing, it was shown that the integrated diaper showed improved wicking performance, reduced sag, and improved discretion.

There is a strong opportunity to improve adult diaper performance using the proposed technologies in this paper. These include a self-shaping liquid activated absorbent core to reduce sag and conform to the body, an origami-inspired wicking layer with improved wicking performance, and a deployable compliant waistband to improve ease of use.

## Data Availability

Data sharing is not applicable to this article as no new data were created or analyzed in this study.
